# Local Action of Moderate Heating and Illumination Induces Electrical Signals, Suppresses Photosynthetic Light Reactions, and Increases Drought Tolerance in Wheat Plants

**DOI:** 10.3390/plants13091173

**Published:** 2024-04-23

**Authors:** Lyubov Yudina, Alyona Popova, Yuriy Zolin, Kseniya Grebneva, Ekaterina Sukhova, Vladimir Sukhov

**Affiliations:** Department of Biophysics, N.I. Lobachevsky State University of Nizhny Novgorod, 603950 Nizhny Novgorod, Russia; lyubovsurova@mail.ru (L.Y.); silverkumiho@mail.ru (A.P.); uchebnayap.zolin@gmail.com (Y.Z.); grebneva.kseniya01@mail.ru (K.G.); n.catherine@inbox.ru (E.S.)

**Keywords:** electrical signals, moderate heating, illumination, photosynthetic regulation, drought tolerance, wheat

## Abstract

Local actions of stressors induce electrical signals (ESs), influencing photosynthetic processes and probably increasing tolerance to adverse factors in higher plants. However, the participation of well-known depolarization ESs (action potentials and variation potentials) in these responses seems to be rare under natural conditions, particularly in the case of variation potentials, which are induced by extreme stressors (e.g., burning). Earlier, we showed that the local action of moderate heating and illumination can induce low-amplitude hyperpolarization ESs influencing photosynthetic light reactions in wheat plants cultivated in a vegetation room. In the current work, we analyzed ESs and changes in photosynthetic light reactions and drought tolerance that were induced by a combination of moderate heating and illumination in wheat plants cultivated under open-ground conditions. It was shown that the local heating and illumination induced low-amplitude ESs, and the type of signal (depolarization or hyperpolarization) was dependent on distance from the irritated zone and wheat age. Induction of depolarization ESs was not accompanied by photosynthetic changes in plants under favorable conditions or under weak drought. In contrast, the changes were observed after induction of these signals under moderate drought. Increasing drought tolerance was also observed in the last case. Thus, low-amplitude ESs can participate in photosynthetic regulation and increase tolerance to drought in plants cultivated under open-ground conditions.

## 1. Introduction

Fast plant adaptation to adverse environmental factors requires induction and propagation of long-distance stress signals providing systemic responses. Electrical signals (ESs), which are transient and propagating changes in an electrical gradient across the plasma membrane, are considered to be important stress signals participating in induction of fast physiological changes in plants [1,2,3,4,5,6,7,8,9,10]. It is known that ESs can induce expression of defense genes (e.g., genes of the proteinase inhibitor 1 and 2 [11,12,13] and anti-insect vegetative storage protein 2 [14]) and genes of signaling pathways (e.g., genes of the jasmonate ZIM domain 10 [14]), production of abscisic, jasmonic and salicylic acids [14,15,16,17], activation of respiration [18,19,20], decrease in phloem uploading [21,22], stopping phloem mass flow [23,24,25], changes in transpiration [26,27], suppression of plant growth [28,29], and many other responses.

Photosynthetic dark and light reactions are likely an important target of ESs [2,3,6,8,10], because electrical signals decrease CO_2_ assimilation [30,31,32], mesophyll conductance for CO_2_ [33], and quantum yields of photosystem I and photosystem II (Φ_PSII_) [34,35] and increase the non-photochemical quenching of chlorophyll fluorescence (NPQ) [19,36,37,38] and cyclic electron flow around photosystem I [35]. It is interesting that the photosynthetic inactivation can be also observed at transmission of ESs from one plant to another [38]. Two main mechanisms are considered to participate in induction of the photosynthetic inactivation [10,19]: (i) decreasing the mesophyll conductance followed by suppression of photosynthetic dark reactions and (ii) direct inactivation of photosynthetic light reactions (mainly suppression of the linear electron flow and activation of NPQ). Both mechanisms are based on transient inactivation of H^+^-ATPase in the plasma membrane, which is an important component in the generation of ESs and changes in pH in the apoplast, cytoplasm, stroma, and lumen of chloroplasts [10].

Increasing plant tolerance of adverse factors is likely the final result of ES-induced physiological changes [6,8,10]. It was shown [17,39,40] that induction of ESs decreases damage to plasma membranes and increases residual concentrations of chlorophylls after exposure to excess light and heating. The positive influence of electrical signals on the tolerance of photosynthetic machinery to increase and decrease temperatures can also be observed [41]. In accordance with our previous work (see reviews [8,10]), ES-induced photosynthetic responses are an important mechanism of increasing plant tolerance of adverse factors because they decrease direct damage of the photosynthetic machinery under stress (through decreasing the linear electron flow and increasing the non-photochemical quenching and cyclic electron flow around photosystem I) and stimulate repair in plants (through increasing the ATP content in plant cells).

Thus, ESs and ES-induced photosynthetic responses seem to be an important mechanism of plant adaptation to adverse factors under environmental conditions; however, there are arguments restricting this hypothesis [10]. Most of the described results were found on the basis of investigations of depolarization electrical signals (DESs), which include initial depolarization and further repolarization [8,10]. Action potentials [1,2,42,43,44,45] and variation potentials [2,46,47,48] are well-known DESs.

Action potentials are short-term self-propagating spikes based on the transient activation of Ca^2+^, anions, and outwardly rectifying K+ channels [1,49]. Transient Ca^2+^-dependent inactivation of H^+^-ATPase in the plasma membrane is another mechanism of action potential generation [50]. It is known that action potentials can be induced by weak stimuli (e.g., light [51,52], cooling [44,50], or touch [45,53]); however, this induction is possible after long-term refractory period (hours) excluding the action potential generation in motor plants. This means that action potentials can participate in plant adaptation to adverse factors under only strongly stable and favorable conditions, which are rare in the environment [10].

Variation potentials are long-term and irregular DESs [2,48], and likely to be local electrical responses induced by propagation of non-electrical signals. Propagation of hydraulic waves [46,47,54], transmission of chemical agents called wound substances (maybe H_2_O_2_ or glutamate) [5,37,55,56], or a combination of both processes [57,58,59] are considered non-electrical signals. Non-electrical signals induce the activation of mechanosensitive or ligand-dependent Ca^2+^ channels providing an influx of calcium ions, increased Ca^2+^ concentration in the cytoplasm, and long-term inactivation of H^+^-ATPase in the plasma membrane [2,8,10,46,60]. Variation potentials can be induced under both favorable and stress conditions (e.g., water deficit [61]); however, using damaging stressors (e.g., burning or extreme heating) is often necessary for their induction [10]. This means that variation potentials can participate in plant adaptation to adverse factors under extreme stress only (e.g., thermal damage in wildfire), which are also rare in the environment [10].

Earlier, we suggested [10] that hyperpolarization electrical signals (HESs) (system potentials in accordance with [62,63]), which include initial hyperpolarization and further repolarization, can participate in plant adaptation to adverse environmental factors without noted restrictions. HESs can be induced under action of widespread stressors with moderate intensity (e.g., moderate heating and illumination [64] or weak pressure [65]) and cause photosynthetic inactivation [31,65,66], likely through alkalization of the apoplast [65] and decreasing the mesophyll CO_2_ conductance [10].

The mechanisms of system potentials are discussed. In accordance with our hypothesis [10], system potentials are local electrical responses caused by propagation of non-electrical signals with low amplitudes, which are induced by stressors with moderate intensities. Probably, the signals are hydraulic waves with low amplitudes [65]; however, transmission of low concentrations of the wound substance cannot be fully excluded either. These non-electrical signals induce weak activation of mechanosensitive (or ligand-dependent) Ca^2+^ channels and small increases in concentrations of calcium ions in the cytoplasm [10]. It is known that small increases in Ca^2+^ concentration suppress K^+^ inwardly rectifying channels [67,68] and thereby can induce hyperpolarization [10]. In contrast, actions of damaging stressors and further propagation of non-electrical signals with moderate or high amplitudes induce strong activation of Ca^2+^ channels and large increases in Ca^2+^ concentration in the cytoplasm, which causes inactivation of H^+^-ATPase in the plasma membrane [8], i.e., variation potentials are formed in this case.

Our previous work [64,66], which showed inducing HESs under moderate stressors (mainly combination of heating to 40 °C and illumination) and further forming photosynthetic inactivation, investigated plants cultivated in a vegetation room. ESs, photosynthetic responses, and plant tolerance changes caused by these stressors in plants, which were cultivated in open-ground conditions (i.e., mimicking natural conditions), have not been investigated. Thus, the current work was devoted to investigation of ESs and changes in photosynthetic light reactions and drought tolerance induced by a combination of heating to 40 °C and illumination in wheat plants, which were cultivated in open-ground conditions. Combinations of the moderate heating and illumination were used to compare the current results with our previous investigations [64,66]. Additionally, this combination is a widespread stressor that can be related to the action of sunlight on plant parts.

## 2. Results

### 2.1. Electrical Signals and Changes in Photosynthetic Light Reactions Induced by Moderate Heating and Illumination in Wheat Plants with Irrigation

**Figure 1:**
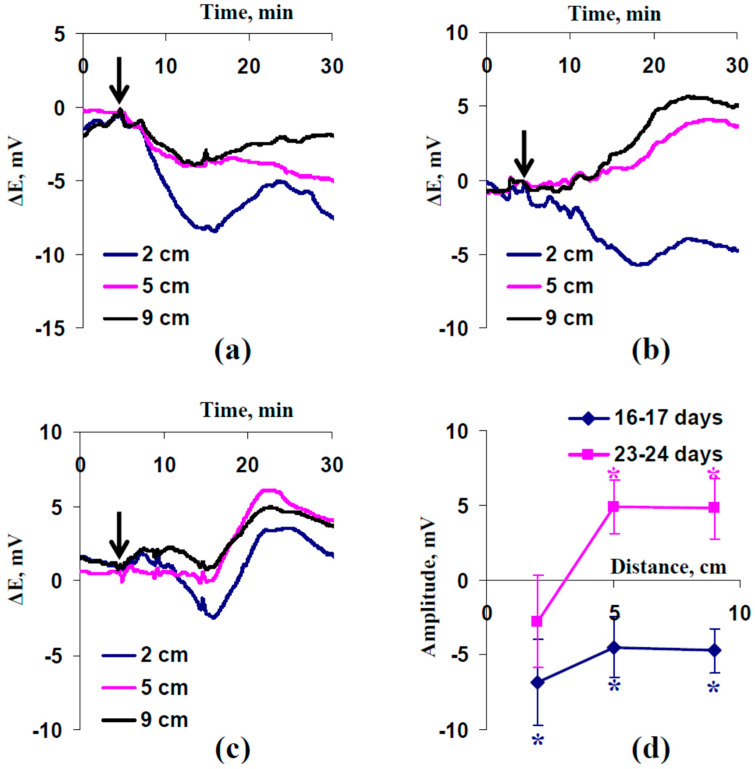
Examples of electrical signals induced in wheat plants after 16–17 (**a**) and 23–24 (**b**,**c**) days of cultivation and average amplitudes of these signals at different distances from the irritated zone (*n* = 10) (**d**). A combination of moderate heating and illumination at the top of the second leaf was used for irritation (arrow). Differences in surface electrical potentials of measuring and reference electrodes (ΔE) were measured; measuring electrodes were placed on the second wheat leaf 2, 5, and 9 cm from the irritated zone. Positive values in ΔE and amplitudes corresponded to hyperpolarization; negative values corresponded to depolarization. * Average amplitude significantly different from zero (*p* < 0.05).

Analysis of ESs induced by the combination of moderate heating and illumination in wheat plants under irrigation was the first stage of the current investigation (Figure 1). It was found that this local irritation induced only depolarization electrical signals with low amplitudes (about 4.5–7 mV) in 16- to 17-day-old wheat plants (Figure 1a,d). However, investigation of plants 23–24 days old showed that both DESs and HESs, which had low amplitudes (about 5 mV), were induced in this case (Figure 1b,d): the depolarization electrical signals were generated 2 cm from the irritated zone, while hyperpolarization electrical signals were generated 5 and 9 cm from this zone. It is interesting that multiphase ESs could also be observed in wheat plants aged 23–24 days (Figure 1c). In this case, ESs, which were generated 2 cm from the irritated zone, had both a depolarization component and a hyperpolarization component in changes in surface potential. In contrast, HESs were mainly observed at increased distances from the zone of action of moderate heating and illumination (5 and 9 cm).

Analysis of average amplitudes of ESs (Figure 1d) showed that absolute values of DESs were weakly decreased with increased distance from the irritated zone in 16- to 17-day-old wheat plants. In 23- to 24-day-old wheat plants, the average amplitude of ESs 2 cm from the irritated zone was low and insignificant (direction of ESs was varied), but the amplitudes of HESs were significant 5 and 9 cm from this zone.

Thus, the combination of heating to 40 °C and illumination induced ESs with low amplitudes in wheat plants cultivated in open-ground conditions. The type of the electrical signals (DESs or HESs) was dependent on the plant age and distance from the irritated zone.

**Figure 2:**
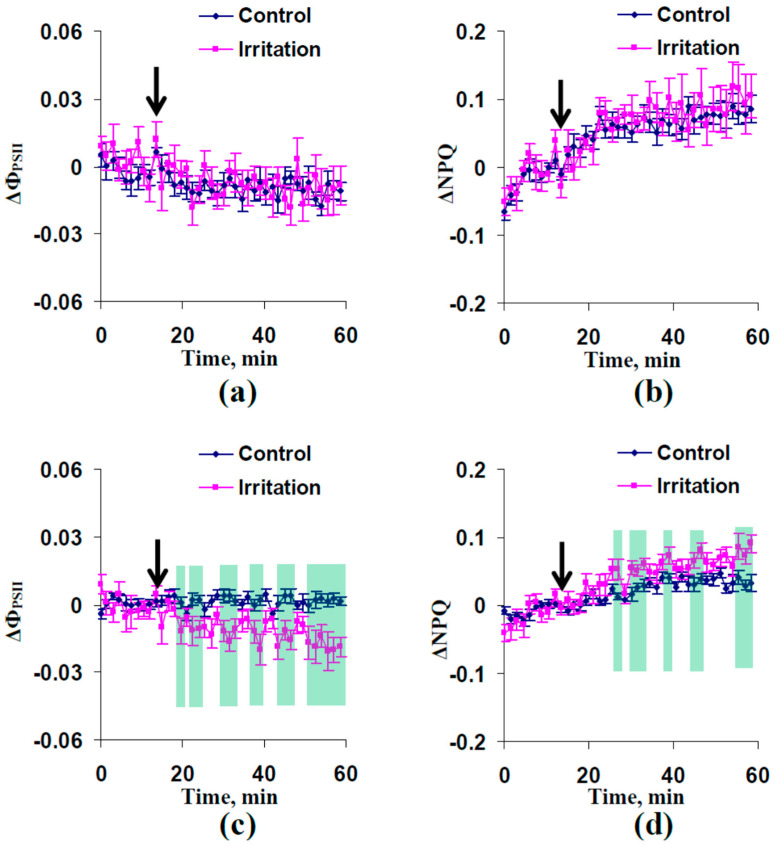
Dynamics of average changes in the quantum yield of photosystem II (ΔΦ_PSII_) and non-photochemical quenching of chlorophyll fluorescence (ΔNPQ) in non-irritated (control) and irritated wheat plants (*n* = 8). (**a**) Dynamics of ΔΦ_PSII_ in plants after 16–17 days of cultivation. (**b**) Dynamics of ΔNPQ in plants after 16–17 days of cultivation. (**c**) Dynamics of ΔΦ_PSII_ in plants after 23–24 days of cultivation. (**d**) Dynamics of ΔNPQ in plants after 23–24 days of cultivation. Combination of moderate heating and illumination at the top of the second leaf was used for irritation (arrow). Photosynthetic parameters were measured 3 cm from the irritated zone. ΔΦ_PSII_ and ΔNPQ were calculated as differences between current values of parameters and these values before the local irritation. Green shading shows significant differences between the experimental and control values (*p* < 0.05).

The influence of the local irritation and subsequent ESs on photosynthetic light reactions was investigated in the next stage. Figure 2 shows changes in Φ_PSII_ (ΔΦ_PSII_) and NPQ (ΔNPQ) in non-irritated parts of leaf that were induced by the combination of the moderate heating and illumination. It was found that significant differences between control (without the local irritation) and experimental (with the local irritation) dynamics of ΔΦ_PSII_ (Figure 2a) and ΔNPQ (Figure 2b) were absent in 16- to 17-day-old wheat plants. In contrast, the moderate heating and illumination decreased the quantum yield of photosystem II (Figure 2c) and increased the non-photochemical quenching (Figure 2d) in the 23- to 24-day-old wheat plants. It was interesting that changes in Φ_PSII_ and NPQ were formed in experimental cases with induction of HESs (23- to 24-day-old plants). In contrast, these changes were absent in cases with induction of only DESs (16- to 17-day-old plants).

Significant differences between control and experimental dynamics of investigated parameters of the photosynthetic light reactions were absent 5, 7, and 9 cm from the irritated zone (Figures S1–S4, Supplementary Materials).

Thus, the moderate stressors (combination of heating to 40 °C and illumination) caused photosynthetic inactivation in 23- to 24-day-old plants, which were cultivated in open-ground conditions and well irrigated (DESs and HESs were both induced in this case), but these photosynthetic changes were absent in the 16- to 17-day-old plants (DESs only were induced).

### 2.2. Electrical Signals and Changes in Photosynthetic Light Reactions Induced by Moderate Heating and Illumination in Wheat Plants under Drought

Our earlier studies [64,66] showed that drought modified parameters of ESs with low amplitudes caused by moderate heating and illumination and strongly influenced characteristics of ES-induced responses of photosynthetic light reactions in wheat plants cultivated in a vegetation room. As a result, analysis of electrical signals and changes in photosynthetic light reactions induced by moderate heating and illumination in wheat plants under drought (open-ground conditions) was performed in the next stage of the current work.

**Figure 3:**
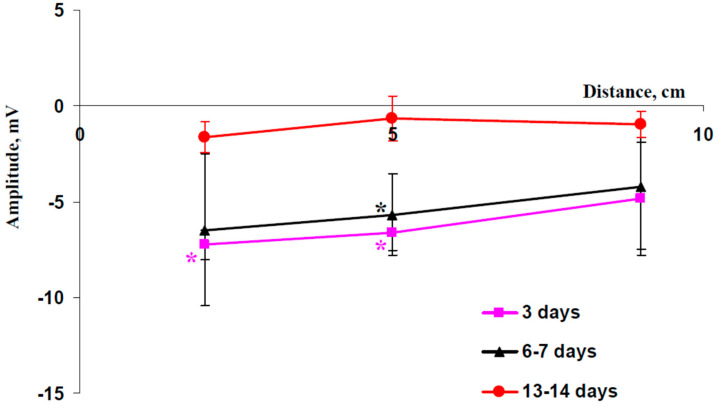
Average amplitudes of electrical signals at different distances from the irritated zone in wheat plants after 3, 6–7, and 13–14 days of drought (*n* = 5–9). A combination of moderate heating and illumination at the top of the second leaf was used for irritation. Electrical signals were measured in the second wheat leaf 2, 5, and 9 cm from the irritated zone. Negative values of amplitudes corresponded to depolarization. Drought was induced by termination of irrigation. Plants were cultivated for 14 days before initiation of the drought. * Average value of amplitude significantly different from zero (*p* < 0.05).

It was found (Figure 3) that the weak (3 days without irrigation) and moderate (6–7 days) drought slightly increased amplitudes of ESs (about 5–7 mV) in comparison with amplitudes of signals induced in the wheat plants under irrigation (see Figure 1d). These ESs were depolarization electrical signals. In contrast, strong drought (13–14 days) suppressed formation of DESs in wheat plants.

Analysis of irritation-induced responses of photosynthetic light reactions showed that these responses were weak after 3 days of drought (Figures S5 and S6). Significant decreases in Φ_PSII_ and significant increases in NPQ were induced only at 5 cm from the irritated zone for a few time points. It should be noted that these minor responses were different from the full absence of significant photosynthetic changes in wheat plants of the same age, which were cultivated with irrigation (Figure 2, Figures S1 and S2).

**Figure 4:**
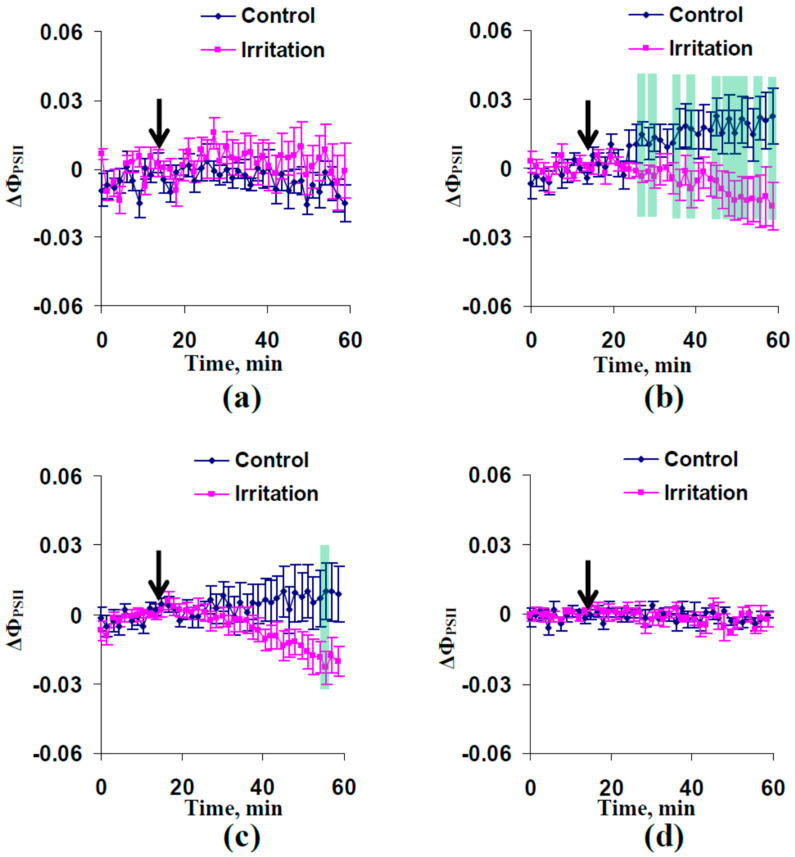
Dynamics of average changes in the quantum yield of photosystem II (ΔΦ_PSII_) 3 (**a**), 5 (**b**), 7 (**c**), and 9 (**d**) cm distances from the irritated zone in wheat plants after 6–7 days of drought (*n* = 12). Combination of moderate heating and illumination at the top of the second leaf was used for irritation (arrow). Drought was induced by termination of irrigation. Plants were cultivated for 14 days before initiation of the drought. Green shading shows significant differences between the experimental and control values (*p* < 0.05).

**Figure 5:**
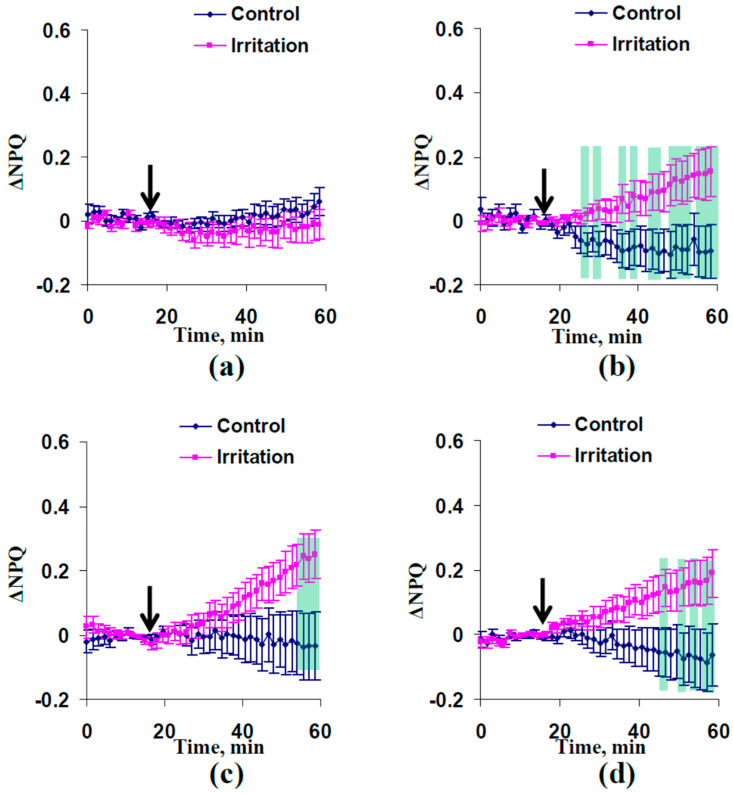
Dynamics of average changes in the non-photochemical quenching of chlorophyll fluorescence (ΔNPQ) 3 (**a**), 5 (**b**), 7 (**c**), and 9 (**d**) cm distances from the irritated zone in wheat plants after 6–7 days of drought (*n* = 12). A combination of moderate heating and illumination at the top of the second leaf was used for irritation (arrow). Drought was induced by termination of irrigation. Plants were cultivated for 14 days before initiation of the drought. Green shading shows significant differences between the experimental and control values (*p* < 0.05).

The moderate drought (6–7 days without irrigation) strongly contributed to induction of photosynthetic responses. It was found (Figure 4) that the combination of moderate heating and illumination caused significantly decreased quantum yield of photosystem II 5 and 7 cm from the irritated zone. Significant increases of NPQ were observed 5, 7, and 9 cm from the irritated zone (Figure 5).

The strong drought (13–14 days without irrigation) suppressed photosynthetic responses induced by the local irritation (Figures S7 and S8). Significant differences between photosynthetic parameters in irritated and non-irritated plants were observed in separate time points only and seemed to be statistical artefacts.

Analysis of the drought influence on electrical signals and photosynthetic responses induced by the combination of moderate heating and illumination in wheat plants cultivated in open-ground conditions, showed that the moderate drought (6–7 days) caused weak stimulation of electrical signals because amplitudes of DESs were insignificantly increased and strong stimulation of photosynthetic inactivation because magnitudes of changes in Φ_PSII_ and NPQ were significantly increased and areas of these changes were extended.

### 2.3. Influence of Moderate Heating and Illumination on Drought Tolerance of Wheat Plants

**Figure 6:**
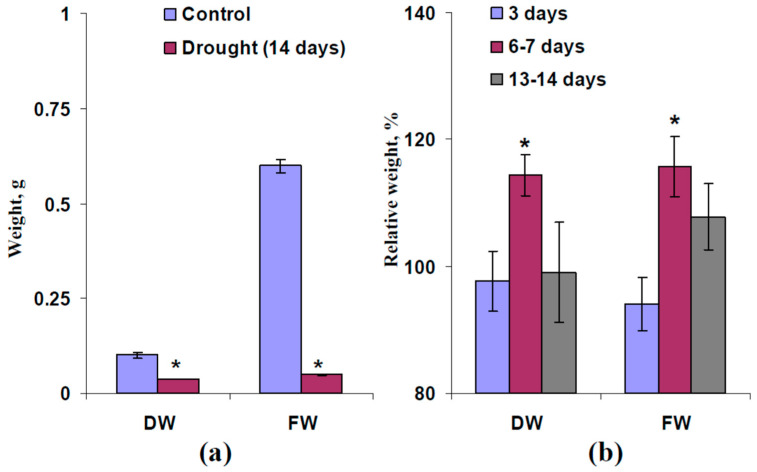
(**a**) Final dry weight (DW) and fresh weight (FW) of wheat shoots in control wheat plants (without drought) and plants after 14 days of drought (*n* = 20–50). Drought was induced by termination of irrigation. Plants were cultivated for 14 days before initiation of the drought. * Significantly different from control (*p* < 0.05). (**b**) Relative values of the final DW and FW (after 14 days of drought) in wheat plants that were irritated after 3 days, 6–7 days, and 13–14 days of drought (*n* = 7–15). Combination of local moderate heating and illumination at the top of the second leaf was used for irritation. The final DW and FW (after 14 days of drought) in non-irritated plants were assumed to be 100%. * Significantly different from 100% (*p* < 0.05).

In the last stage of the current investigation, we analyzed influence of moderate heating and illumination on the plant tolerance of drought. It was shown that drought induced strong decreases in dry wight (DW) and fresh weight (FW) of shoots, which were measured after 14 days without irrigation in wheat plants (open-ground conditions) (Figure 6a). This result showed plant damage by drought.

We analyzed final relative DW and FW (after 14 days of drought) to accurately estimate the influence of the irritation used on plant tolerance. It was shown (Figure 6b) that moderate heating and illumination of plants after 3 days or 13–14 days of drought did not influence the final DW and FW. In contrast, the local irritation in wheat plants after 6–7 days of drought significantly increased the final DW and FW.

**Figure 7:**
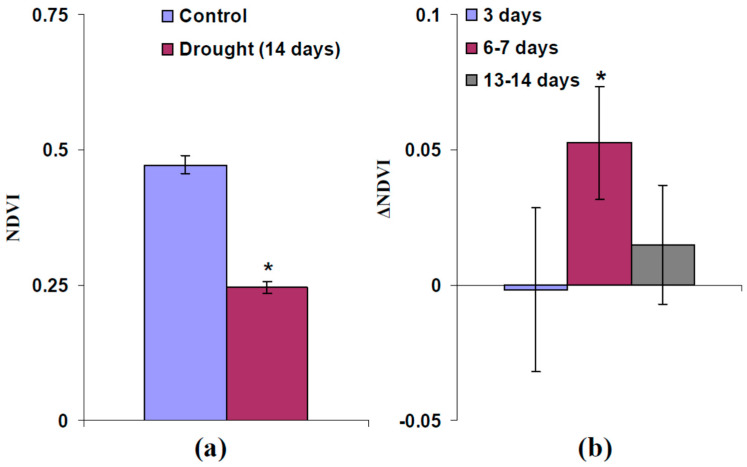
(**a**) Final normalized difference vegetation index (NDVI) in control wheat plants (without drought) and plants after 14 days of drought (*n* = 96–120). Drought was induced by termination of irrigation. Plants were cultivated for 14 days before initiation of drought. * Significantly different from control (*p* < 0.05). (**b**) Irritation-induced changes in final NDVI after 14 days of drought (ΔNDVI) in the wheat plants (*n* = 7–15). Combination of local moderate heating and illumination at the top of the second leaf was used for irritation. Wheat plants were irritated after 3 days, 6–7 days, and 13–14 days of drought. ΔNDVI was calculated as difference between the final NDVI in irritated and non-irritated plants. * Significantly different from zero (*p* < 0.05).

Changes in the final normalized difference vegetation index (NDVI), which was measured after 14 days of drought, were similar: strong drought provided low final NDVI (Figure 7a); however, moderate heating and illumination after 6–7 days of drought increased this parameter (Figure 7b).

Thus, the results showed a positive influence of the combination of the moderate heating and illumination after 6–7 days of drought on wheat plant tolerance of drought action. It was interesting that this positive effect was observed after large photosynthetic light reactions (decreasing Φ_PSII_ and increasing NPQ). Weak photosynthetic responses, which were observed after 3 days and 13–14 days of drought, were not accompanied by changes in plant tolerance of drought.

## 3. Discussion

Electrical signals influence numerous physiological processes in higher plants (including photosynthesis) and can increase their tolerance of adverse factors [1,2,3,4,5,6,7,8,9,10]. As a result, electrical signals are a potential mechanism for fast plant adaptation to changeable environmental conditions [8,69]. However, induction of well-known DESs can be restricted under natural conditions [10] because action potentials are mainly propagated under stable and favorable environmental characteristics and variation potentials are caused by relatively rare damaging stressors (e.g., extreme heating or burning related to wildfires).

Our earlier results [10,64,65,66] show that HESs with low amplitudes (probably system potentials, in accordance with [62,63]) can be electrical signals participating in this fast plant adaptation. This hypothesis is supported by (i) induction of hyperpolarization electrical signals under moderate irritations (e.g., heating to 40 °C, illumination, combination of these stressors, or increased pressure [64,65,66]), which is often observed under natural conditions [10]; (ii) the HES-dependent inactivation of photosynthesis [31,64,66], which is similar to DES-induced photosynthetic responses [30,31,32,33,34,35,36,37] and can potentially positively influence plant tolerance of adverse factors [10,69]; and (iii) stimulation of this photosynthetic inactivation under moderate drought [66]. This stimulation (increasing magnitude and extending region of photosynthetic changes) can also contribute to plant adaptation to adverse factors (e.g., water deficit).

However, earlier investigations of HESs were mainly based on plants cultivated under the controlled conditions of a vegetation room [64,65,66]. This means that their results can be limitedly relevant for plants, which are cultivated under natural conditions. Additionally, the influence of ESs with low amplitudes on plant tolerance of adverse factors (including drought) were not investigated in our previous work. Results of the current work, which are devoted to investigation of wheat plants cultivated in open-ground conditions and analysis of changes in plant drought tolerance, clarify these points.

First, it is shown that a combination of moderate heating and illumination can induce both HESs and DESs under favorable conditions (Figure 1): both variants of ES have low amplitudes (less than 10 mV). The effect is dependent on plant age: only DESs are observed in 16- to 17-day-old wheat plants, and both DESs (near the irritated zone) and HESs (at a distance from this zone) are generated in 23- to 24-day-old plants. These results are in good accordance with the strong relationships between DESs (variation potentials) and HESs (system potentials) found in other studies [61,63,64,65].

These relationships are probably based on a key role of Ca^2+^ influx in induction of both DESs and HESs [10]: weak Ca^2+^ influx inactivates inwardly rectifying K^+^ channels in the plasma membrane [67,68] and induces hyperpolarization (system potentials), and moderate or strong Ca^2+^ influx inactivates H^+^-ATPase in the plasma membrane [70] and induces depolarization (variation potentials). The amplitude of non-electrical signals (increasing pressure [46,47,54] or maybe increasing concentration of the wound substance [5,37,55,56]), which is dependent on characteristics of the local irritation and parameters of the plant, seems to determine the type of ES [10,65]. It is additionally interesting that our current results show multiphase ESs (Figure 1c), in good accordance with theory (transformation of DESs into HESs with increasing distance from the irritated zone [10]) and with multiphase ESs shown under local increased pressure [65].

Second, induction of ESs with low amplitudes can be accompanied by the formation photosynthetic responses (decreasing Φ_PSII_ and increasing NPQ) in wheat plants under irrigation; however, relationships of ESs and photosynthetic changes are intricate (Figure 2). In particular, propagation of both DESs and HESs (Figure 1b) in 23- to 24-day-old plants is related to induction of responses of photosynthetic light reactions (Figure 2c,d). This result is in good accordance with our previous work [65,66] showing decreasing Φ_PSII_ and increasing NPQ after moderate irritation. The apoplastic alkalization accompanying HESs [62] and related to the decrease in relative activity of H^+^-ATPase [10] is a probable mechanism of disruption of the CO_2_ influx (as a result of changes in CO_2_/HCO_3_^−^ ratio influencing CO_2_ transport across the plasma membrane) and suppression of photosynthetic processes [10].

In contrast, photosynthetic changes were not observed (Figure 2a,b) in 16- to 17-day-old plants, which were characterized by only DESs with low amplitudes (Figure 1a). It is known that DESs (at least, action potentials and variation potentials with high amplitudes of tens of mV) are also accompanied by alkalization of the apoplast [34,49,50,60] and suppress photosynthetic processes [30,31,32,33,34,35,36,37]. Potentially, the absence of photosynthetic changes under the only DES propagation in the current work can be related to low amplitudes of DESs (Figure 1d) because magnitudes of pH changes are decreased with decreasing amplitudes of ESs [60]. However, large changes in the apoplastic pH (up to about 0.5) can be observed during even electrical signals with low amplitudes [60]. This means that DESs can potentially induce photosynthetic responses under more suitable experimental conditions.

Earlier, we showed that moderate drought increases magnitudes of HES-induced changes in Φ_PSII_ and NPQ and extends the region of these changes [66]. It can be expected that similar effects should be observed in the current work. Our results show that weak (3 days) and moderate (6–7 days) drought does not significantly influence parameters of DESs in wheat plants cultivated under open-ground conditions (Figure 1 and Figure 3). However, weak irritation-induced photosynthetic responses (decreasing Φ_PSII_ and increasing NPQ) were observed after 3 days of drought (Figures S5 and S6) and large irritation-induced photosynthetic responses were formed after 6–7 days of drought (Figure 4 and Figure 5). These results show that DESs with low amplitudes induced by a combination of moderate heating and illumination can also cause photosynthetic responses in wheat plants, i.e., the influence of HESs and DESs with low amplitudes on photosynthesis seems to be similar. Strong drought (13–14 days) suppresses both DESs (Figure 3) and eliminates changes in photosynthetic light reactions (Figures S7 and S8). This result is in good accordance with other work [66] and can be explained by extensive damage to wheat plants under these conditions (e.g., Figure 6a shows decreasing DW of wheat shoot from 0.101 to 0.037 g after 14 days of drought).

Third, on the basis of research devoted to the influence of action potentials and variation potentials on photosynthesis [15,16,19,30,31,32,33,34,35,36,37], we earlier hypothesized (see, e.g., reviews [8,10,69]) that ES-induced photosynthetic responses are an important mechanism in increasing plant tolerance of adverse factors (including the photosynthetic machinery tolerance). ES-induced increasing plant tolerance has been shown in numerous studies [17,39,40,41,71,72]. There are several potential modes of influence of ES-induced photosynthetic responses on plant tolerance [8,10,69]: (i) decreasing direct photodamage of the photosynthetic machinery through stimulation of NPQ [73,74,75], (ii) restricting production of reactive oxygen species through stimulation of NPQ and the cyclic electron flow around photosystem I and suppression of the linear electron flow [76,77], which contributes to decreasing damage to photosynthetic machinery and other structures in plant cells, and (iii) increasing the ATP content in the mesophyll cells through suppression of photosynthetic dark reactions [78], which contributes to the repair of the photosynthetic machinery [79] and probably other cell structures. Our current results show that at least decreasing the linear electron flow (on the basis of decreasing Φ_PSII_, Figure 2c and Figure 4) and increasing NPQ (Figure 2d and Figure 5), which are induced by moderate heating and illumination, can contribute to plant tolerance of adverse factors.

Further results of the current work show that induction of DESs with low amplitudes in wheat plants after 6–7 days of drought, which is accompanied by photosynthetic responses (Figure 4 and Figure 5), increases the final DW and FW of shoots (Figure 6) and the final NDVI (Figure 7), which is related to content of chlorophylls [80], after 14 days of drought. In contrast, induction of DESs after 3 days and 13–14 days of drought, which is not accompanied by large photosynthetic responses (Figures S5–S8), does not influence the final DW, FW, or NDVI (Figure 6 and Figure 7). This means that photosynthetic responses, which accompany DESs, contribute the total tolerance (the final biomass after drought is increased) and the tolerance of photosynthetic machinery (the final chlorophyll content, which is estimated on basis of NDVI, is increased) in wheat plants cultivated under open-ground conditions; i.e., they can participate in plant adaptation to adverse factors under natural conditions.

Our previous research, which investigated wheat plants cultivated in a vegetation room [64,65,66], and the current work, which investigated plants cultivated under open-ground conditions, show that ESs with low amplitudes can be induced by irritation with moderate intensities (e.g., combination of heating to 40 °C and illumination). These ESs can include both DESs and HESs, and the type of signal is dependent on the distance from the irritated zone, wheat age, and growth conditions (a vegetation room or open ground). Induction of ESs with low amplitudes (especially HESs) can be accompanied by changes in photosynthetic light reactions. Moderate drought increases the magnitudes of these responses and extends the region of their localization. Induction of ESs with low amplitudes after moderate drought increases plant drought tolerance, and this increase can be caused by photosynthetic responses.

Finally, it should be noted that we did not separately analyze responses, which are induced by moderate heating only and/or illumination only, in the current work. This was for two reasons: necessity of comparison of the current results with our previous results [64,66], which are mainly based on investigation of the combination of moderate heating and illumination and wide dissemination of the combination of heating and illumination under natural conditions (e.g., under sunlight). However, it can be supposed that moderate heating can play a key role in the induction of ESs and photosynthetic inactivation, because illumination induces only small ESs [64] and weakly influences photosynthetic parameters [66] in plants cultivated in a vegetation room.

## 4. Materials and Methods

### 4.1. Plant Material and Cultivation

Spring wheat plants (*Triticum aestivum* L., cultivar Daria) were used as the plant material in the current investigation. Wheat plants were cultivated in vegetation pots with universal soil (300 g soil per pot) in open-ground conditions (Russia, Nizhny Novgorod, July, duration of daylight was 18 h, average daytime temperature 24 °C, average nighttime temperature 16 °C). Each pot contained four wheat plants (two plants were locally irritated and two plants were not locally irritated during experiments). Both irritated and non-irritated plants in the same pot had similar irrigation conditions (well irrigated or development of drought). Plants were irrigated by water every two days; natural irrigation was excluded. Wheat plants 16–17 days old and 23–24 days old were investigated in experiments under the irrigation conditions.

### 4.2. Drought Induction and Estimation of DW, FW, and RWC of Wheat Shoots

Drought was induced by termination of irrigation after 14 days of wheat cultivation under favorable conditions. The total duration of drought was 14 days. Measurements of ESs and parameters of photosynthetic light reactions were performed after 3, 6–7, and 13–14 days of drought.

The final dry weight (DW) and fresh weight (FW) of wheat shoots were measured after 14 days of drought (experiment) or after 14 days of irrigation (control). DW was measured after 2 h treatment of samples with high temperature (about 100 °C) in a TV-20-PZ-K thermostat (Kasimov Instrument Plant, Kasimov, Russia), in accordance with [64,66].

**Figure 8:**
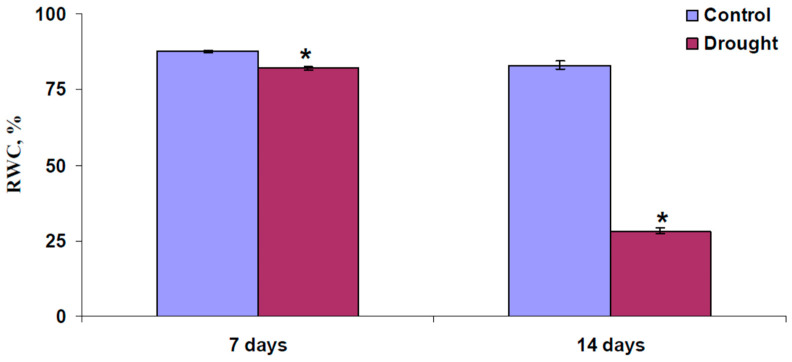
Relative water content (RWC) in wheat shoots after 7 and 14 days of drought (*n* = 20–50). Drought was induced by termination of irrigation; control plants were irrigated. * Significantly different from control (*p* < 0.05).

RWC was calculated as 100%·(FW-DW)/FW. Figure 8 shows the RWC in wheat shoots after 7 and 14 days of drought (without irrigation). It was found that both 7 days of drought and 14 days of drought induced significant decreases in RWC; however, the magnitude of this decrease after 14 days of drought was much larger.

The final relative DW and FW were calculated as 100%·DW_i_/DW_ni_ and 100%·FW_i_/FW_ni_, respectively, where DW_i_ and DW_ni_ were dry weights of shoots of the irritated and non-irritated wheat plants and FW_i_ and FW_ni_ were fresh weights of shoots of the irritated and non-irritated wheat plants. The final relative DW and FW were separately calculated for each pot. After that, they were averaged.

### 4.3. Plant Local Irritation and Measurements of Electrical Signals

The combination of heating to 40 °C and illumination (blue light, 540 µmol m^−2^s^−1^) at the top of the second leaf (about 4 cm) of a wheat plant was used as the local irritation, in accordance with [64,66]. The local irritation was initiated after 60-min plant adaptation in the measuring system.

The self-manufactured system, including blue light LED TDS-P003L4C04, 460 nm, 40 lm, 3 W (TDS Lighting Co., Wuxi, China) and a Peltier element STORM-71, 3.6 A, 36 W (Kryotherm, St. Petersburg, Russia), was used for illumination and heating. The LED was equipped with a black tube to prevent illumination of other parts of the plant. Intensities of light and leaf temperature were measured by a light flux meter PM100D with sensor S120C (Thorlabs Ultrafast Optoelectronics, Ann Arbor, Michigan, United States) and thermometer monitor ATE-9380 (Aktakom, Moscow, Russia), respectively. In accordance with [64,66], heating and illumination were simultaneously initiated, and the duration of the light was 10 min. Local heating continued until the termination of the experiment.

The system—extracellular Ag^+^/AgCl electrodes (RUE Gomel Measuring Equipment Plant, Gomel, Belarus) and a high-impedance IPL-113 amplifier (Semico, Novosibirsk, Russia)—and a personal computer were used for measurements of surface electrical potentials. There were three measuring electrodes, placed 2, 5, and 9 cm from the border of the irritated zone, and the reference electrode was placed on the base of the shoot (near ground level). Dynamics of differences in surface electrical potentials of measuring and reference electrodes (ΔE) were measured for each distance from the irritated zone (2, 5, and 9 cm).

### 4.4. Measurements of Parameters of Photosynthetic Light Reactions

The PAM imaging Open FluorCam FC 800-O/1010 (Photon Systems Instruments, Drasov, Czech Republic) was used for measuring Φ_PSII_ and NPQ in the second leaf of wheat plants. Four wheat plants from the same pot were simultaneously measured.

The maximum fluorescence (Fm) [81] was measured using the standard saturation pulse of this system (4000 μmol m^−2^ s^−1^, cold white light, 6500 K) after 15 min dark adaptation, and the white actinic light (456 µmol m^−2^s^−1^) was turned on. Periodic saturation pulses (every 90 s) were turned on after the 30 min illumination by the actinic light. The maximum fluorescence (Fm’) and steady-state fluorescence (F_t_) under the light conditions were measured using saturation pulses [81]. Two experimental plants were irritated after 15 min of generation of saturation pulses and two control plants were not irritated. The duration of photosynthetic measurements after the local irritation was 45 min.

Spatial distributions of Φ_PSII_ and NPQ were calculated in accordance with standard equations [81] using the software of the Open FluorCam FC 800-O/1010. These photosynthetic parameters were analyzed in four ROIs of the same length (about 0.5 cm) in the second leaf of each wheat plant (in accordance with [65,66]), and centers of ROIs were located 3, 5, 7, and 9 cm from the irritated zone.

ΔΦPSII and ΔNPQ, calculated as Φ_PSII_—Φ_PSII_^0^ and NPQ—NPQ^0^, respectively, were analyzed in the investigation to decrease individual variability in photosynthetic parameters in investigated wheat plants. Φ_PSII_^0^ and NPQ^0^ were Φ_PSII_ and NPQ, which were averaged on the basis of three time points before initiation of the local irritation.

### 4.5. Measurements of NDVI

The handheld PolyPen RP 410 UVIS system (Photon Systems Instruments, Drásov, Czech Republic) was used for measuring the final reflectance spectra of wheat leaves in control plants and in plants after 14 days of drought. The normalized difference vegetation index (NDVI), which is sensitive to concentrations of chlorophylls, was calculated by the software of this system in accordance with a standard equation [80].

### 4.6. Statistics

Quantities of repetitions are given in the captions of the figures. Representative records, averaged records, mean values, and standard errors are presented in the figures. Significance of differences was estimated using Student’s *t*-test. Microsoft Excel 365 (Microsoft Corporation, Redmond, WA, USA) was used for statistical calculations.

## 5. Conclusions

In the current work, we investigated ESs, photosynthetic responses, and changes in plant tolerance to drought, which were induced by a combination of heating to 40 °C and illumination, in wheat plants cultivated in open-ground conditions. First, it was shown that only DESs were induced by local irritation in plants aged 16–17 days. In contrast, both DESs and HESs were observed in plants aged 23–24 days. The weak and moderate drought did not significantly influence the amplitudes of ESs, and the strong drought suppressed electrical signals. Second, induction of DESs and HESs was accompanied by changes in Φ_PSII_ and NPQ in plants aged 23–24 days under irrigation. The moderate drought increased the photosynthetic response and extended the area of changes in Φ_PSII_ and NPQ in wheat leaves. The irritation-induced photosynthetic changes were small under weak and strong drought. Third, induction of ESs with low amplitudes after moderate drought increased plant drought tolerance, most likely through photosynthetic responses.

## Data Availability

The data presented in this study are available upon request from the corresponding author.

## Supplementary Materials

The following supporting information can be downloaded at https://www.mdpi.com/article/10.3390/plants13091173/s1. Figure S1: Dynamics of average changes in the quantum yield of photosystem II (ΔΦ_PSII_) at 3 (a), 5 (b), 7 (c), and 9 (d) cm distances from the irritated zone in wheat plants after 16–17 days of cultivation; Figure S2: Dynamics of average changes in the non-photochemical quenching of chlorophyll fluorescence (ΔNPQ) at 3 (a), 5 (b), 7 (c), and 9 (d) cm distances from the irritated zone in wheat plants after 16–17 days of cultivation; Figure S3: Dynamics of average changes in the quantum yield of photosystem II (ΔΦ_PSII_) at 3 (a), 5 (b), 7 (c), and 9 (d) cm distances from the irritated zone in wheat plants after 23–24 days of cultivation; Figure S4: Dynamics of average changes in the non-photochemical quenching of chlorophyll fluorescence (ΔNPQ) at 3 (a), 5 (b), 7 (c), and 9 (d) cm distances from the irritated zone in wheat plants after 23–24 days of cultivation; Figure S5: Dynamics of average changes in the quantum yield of photosystem II (ΔΦ_PSII_) at 3 (a), 5 (b), 7 (c), and 9 (d) cm distances from the irritated zone in wheat plants after 3 days of drought; Figure S6: Dynamics of average changes in the non-photochemical quenching of chlorophyll fluorescence (ΔNPQ) at 3 (a), 5 (b), 7 (c), and 9 (d) cm distances from the irritated zone in wheat plants after 3 days of drought; Figure S7: Dynamics of average changes in the quantum yield of photosystem II (ΔΦ_PSII_) at 3 (a), 5 (b), 7 (c), and 9 (d) cm distances from the irritated zone in wheat plants after 13–14 days of drought; Figure S8: Dynamics of average changes in the non-photochemical quenching of chlorophyll fluorescence (ΔNPQ) at 3 (a), 5 (b), 7 (c), and 9 (d) cm distances from the irritated zone in wheat plants after 13–14 days of drought.
