# Supplementary material for: Local Action of Moderate Heating and Illumination Induces Electrical Signals, Suppresses Photosynthetic Light Reactions, and Increases Drought Tolerance in Wheat Plants
Source: Plants (Basel). 2024 Apr 23;13(9):1173. doi: 10.3390/plants13091173 (PMC11085084; doi:10.3390/plants13091173)
Supplement: Supplementary file 1 [file plants-13-01173-s001.zip › plants-2961735-supplementary.pdf]

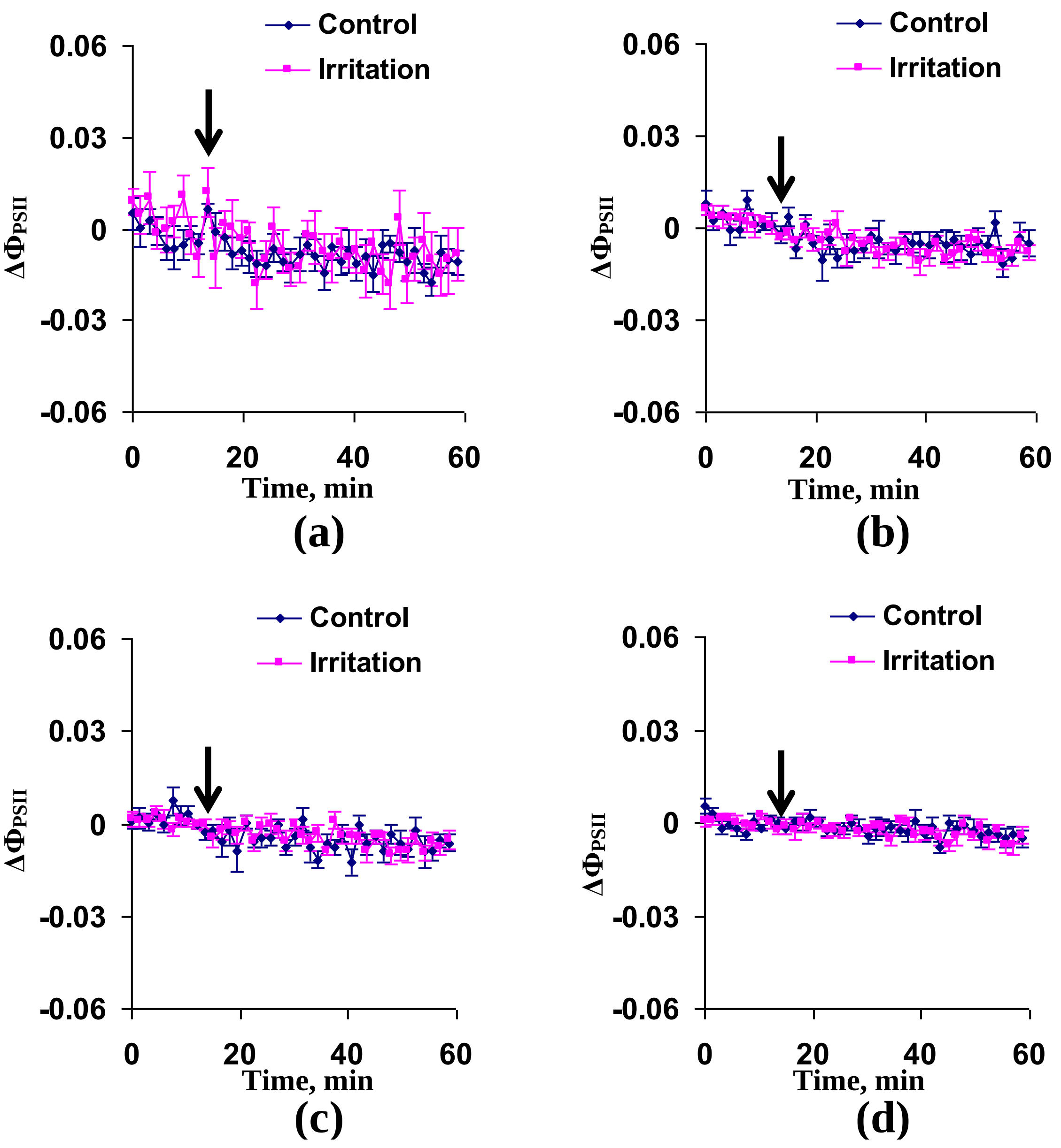

Figure S1. Dynamics of average changes in the quantum yield of photosystem II ($\Delta\Phi_{PSII}$) on 3 **(a)**, 5 **(b)**, 7 **(c)**, and 9 **(d)** cm distances from the irritated zone in wheat plants after 16-17 days of cultivation (n=8). Combination of local moderate heating and illumination of top of the second leaf was used for irritation (arrow). Significant differences between the experimental and control values were absent.

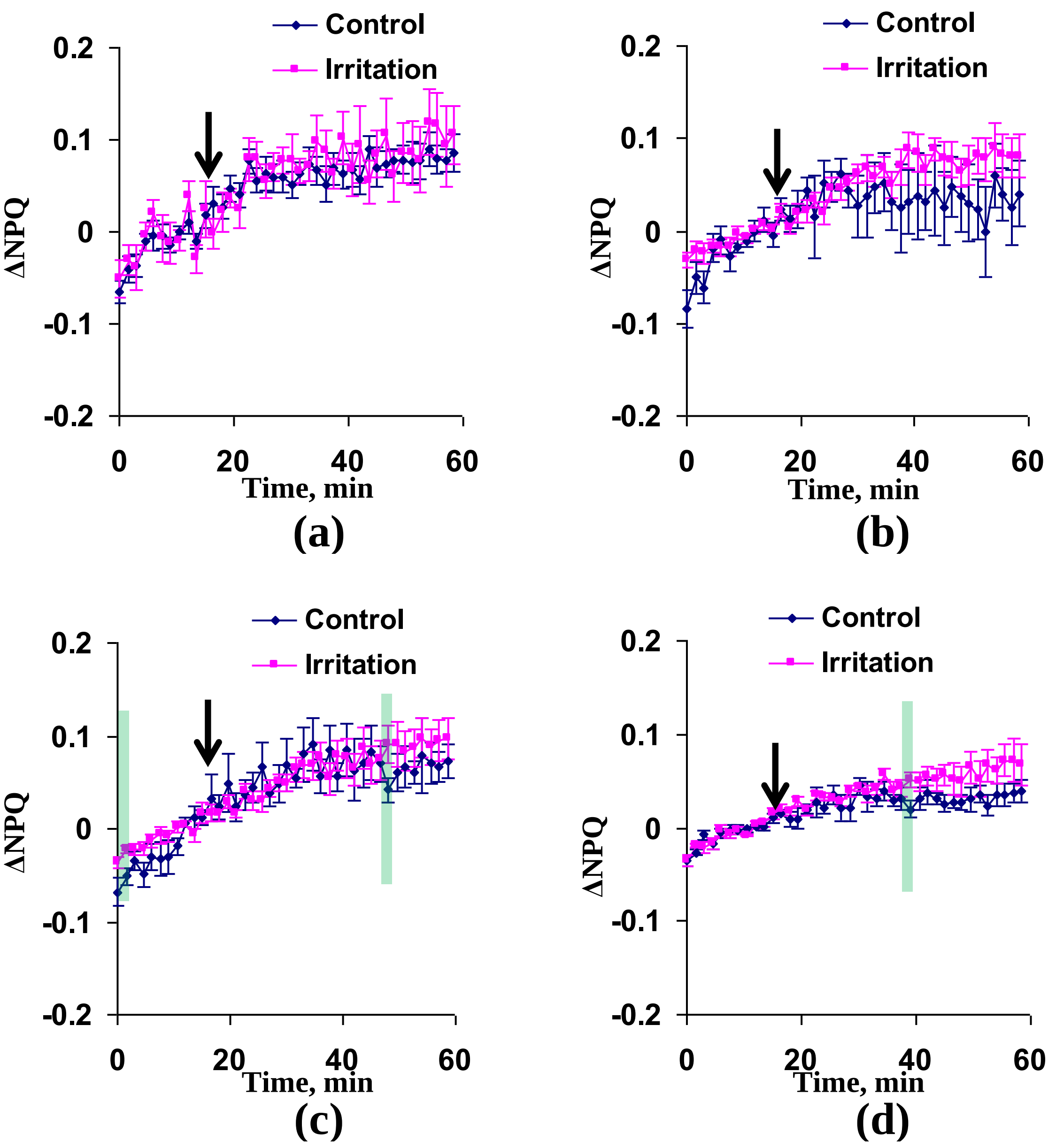

Figure S2. Dynamics of average changes in the non-photochemical quenching of chlorophyll fluorescence (ΔNPQ) on 3 **(a)**, 5 **(b)**, 7 **(c)**, and 9 **(d)** cm distances from the irritated zone in wheat plants after 16-17 days of cultivation (n=8). Combination of local moderate heating and illumination of top of the second leaf was used for irritation (arrow). Green shading shows significant differences between the experimental and control values (p<0.05).

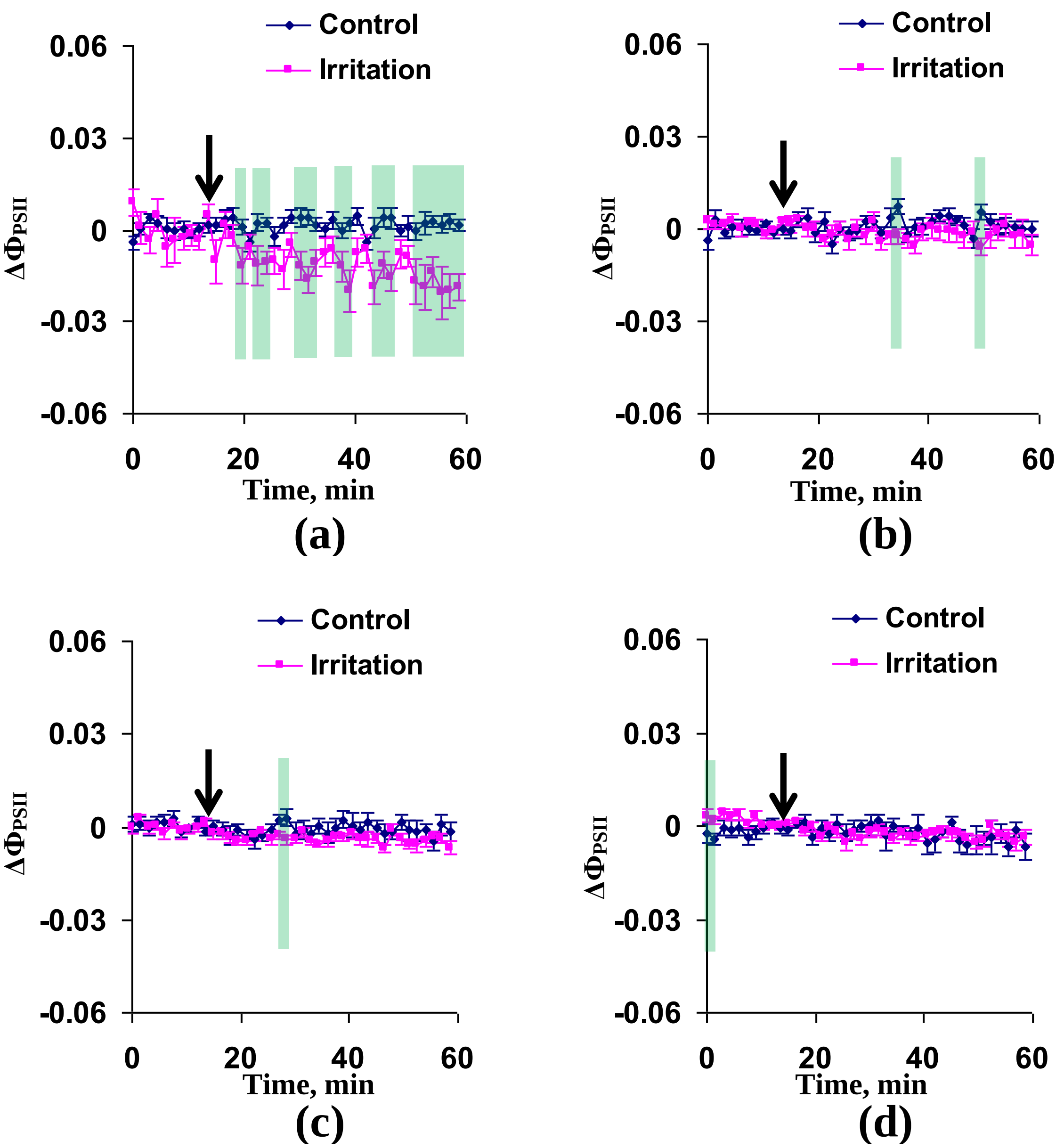

Figure S3. Dynamics of average changes in the quantum yield of photosystem II ($\Delta\Phi_{PSII}$) on 3 **(a)**, 5 **(b)**, 7 **(c)**, and 9 **(d)** cm distances from the irritated zone in wheat plants after 23-24 days of cultivation (n=8). Combination of local moderate heating and illumination of top of the second leaf was used for irritation (arrow). Green shading shows significant differences between the experimental and control values (p<0.05).

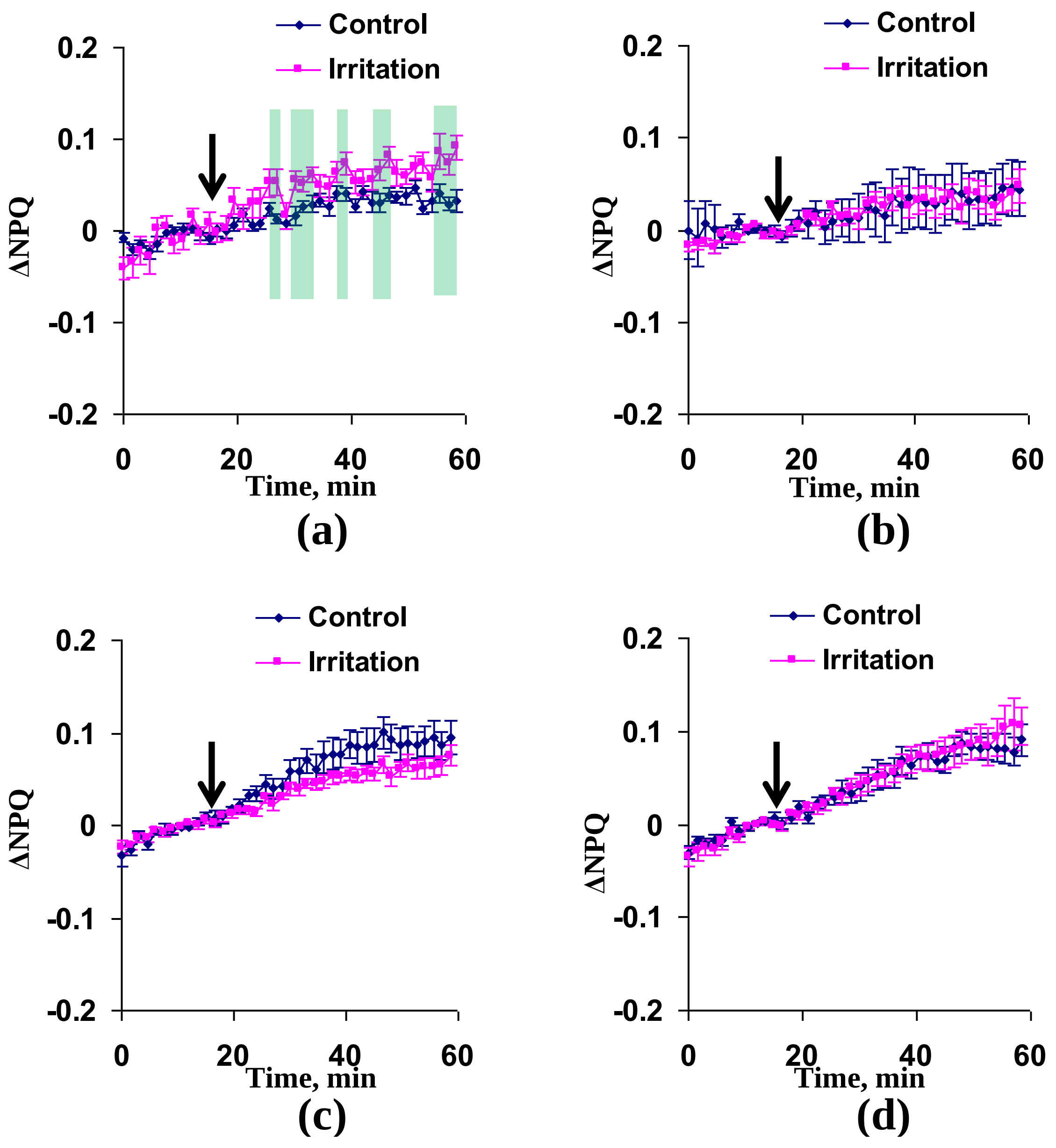

Figure S4. Dynamics of average changes in the non-photochemical quenching of chlorophyll fluorescence (ΔNPQ) on 3 **(a)**, 5 **(b)**, 7 **(c)**, and 9 **(d)** cm distances from the irritated zone in wheat plants after 23-24 days of cultivation (n=8). Combination of local moderate heating and illumination of top of the second leaf was used for irritation (arrow). Green shading shows significant differences between the experimental and control values (p<0.05).

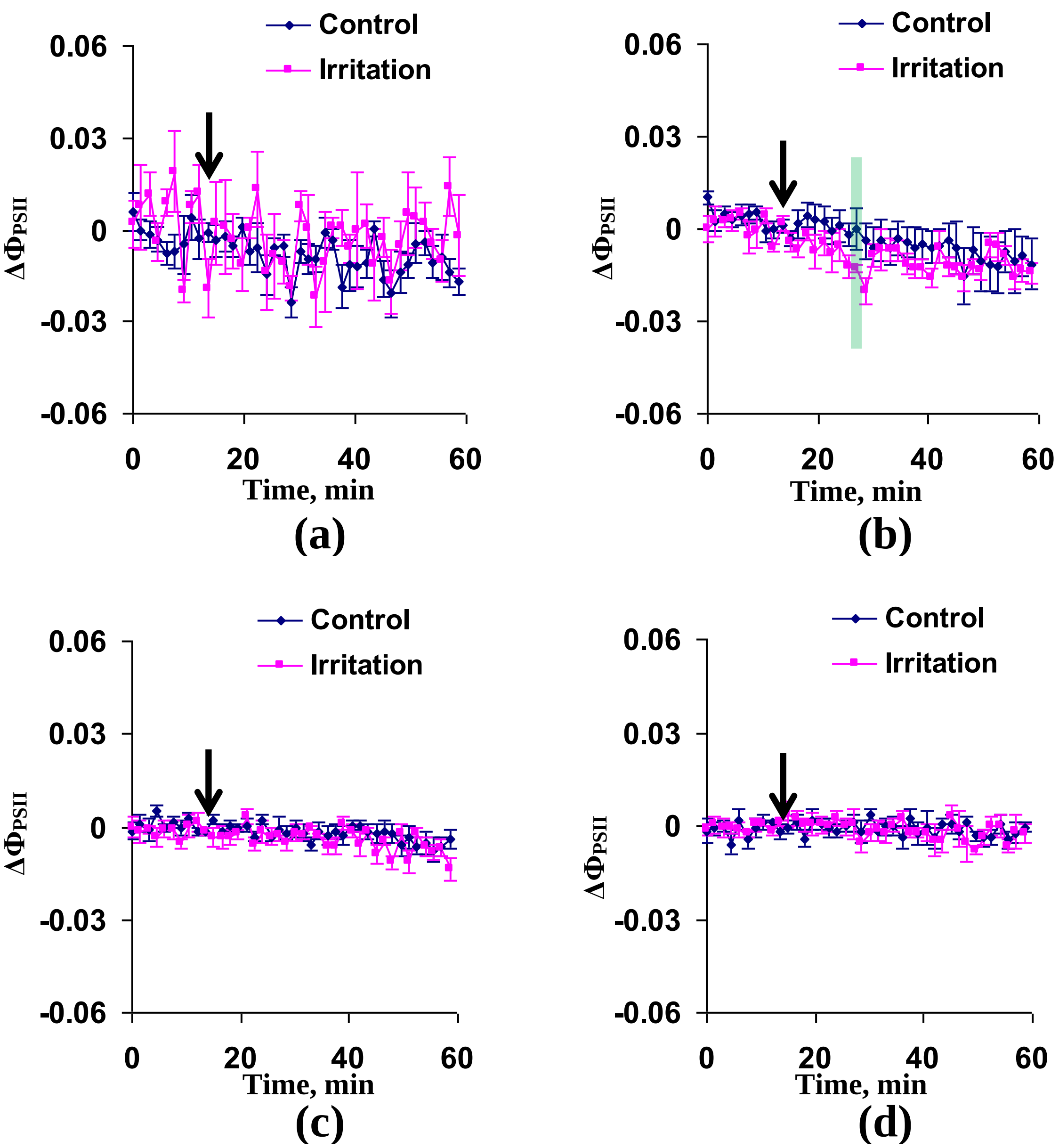

Figure S5. Dynamics of average changes in the quantum yield of photosystem II ($\Delta\Phi_{PSII}$) on 3 **(a)**, 5 **(b)**, 7 **(c)**, and 9 **(d)** cm distances from the irritated zone in wheat plants after 3 days of drought (n=8). Combination of local moderate heating and illumination of top of the second leaf was used for irritation (arrow). Drought was induced by termination of irrigation. Plants were cultivated for 14 days before initiation of the drought. Green shading shows significant differences between the experimental and control values (p<0.05).

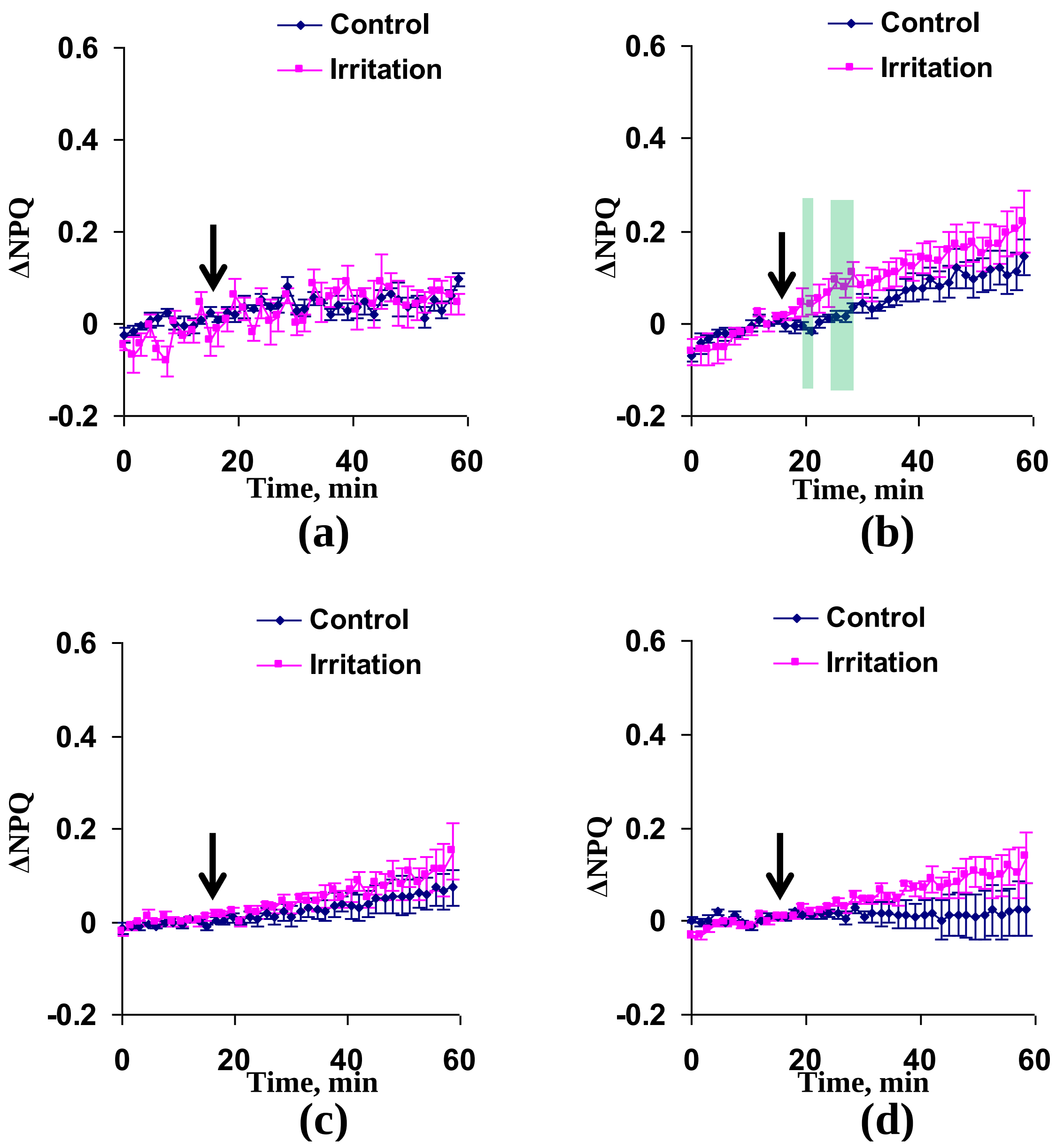

Figure S6. Dynamics of average changes in the non-photochemical quenching of chlorophyll fluorescence (ΔNPQ) on 3 **(a)**, 5 **(b)**, 7 **(c)**, and 9 **(d)** cm distances from the irritated zone in wheat plants after 3 days of drought (n=8). Combination of local moderate heating and illumination of top of the second leaf was used for irritation (arrow). Drought was induced by termination of irrigation. Plants were cultivated for 14 days before initiation of the drought. Green shading shows significant differences between the experimental and control values (p<0.05).

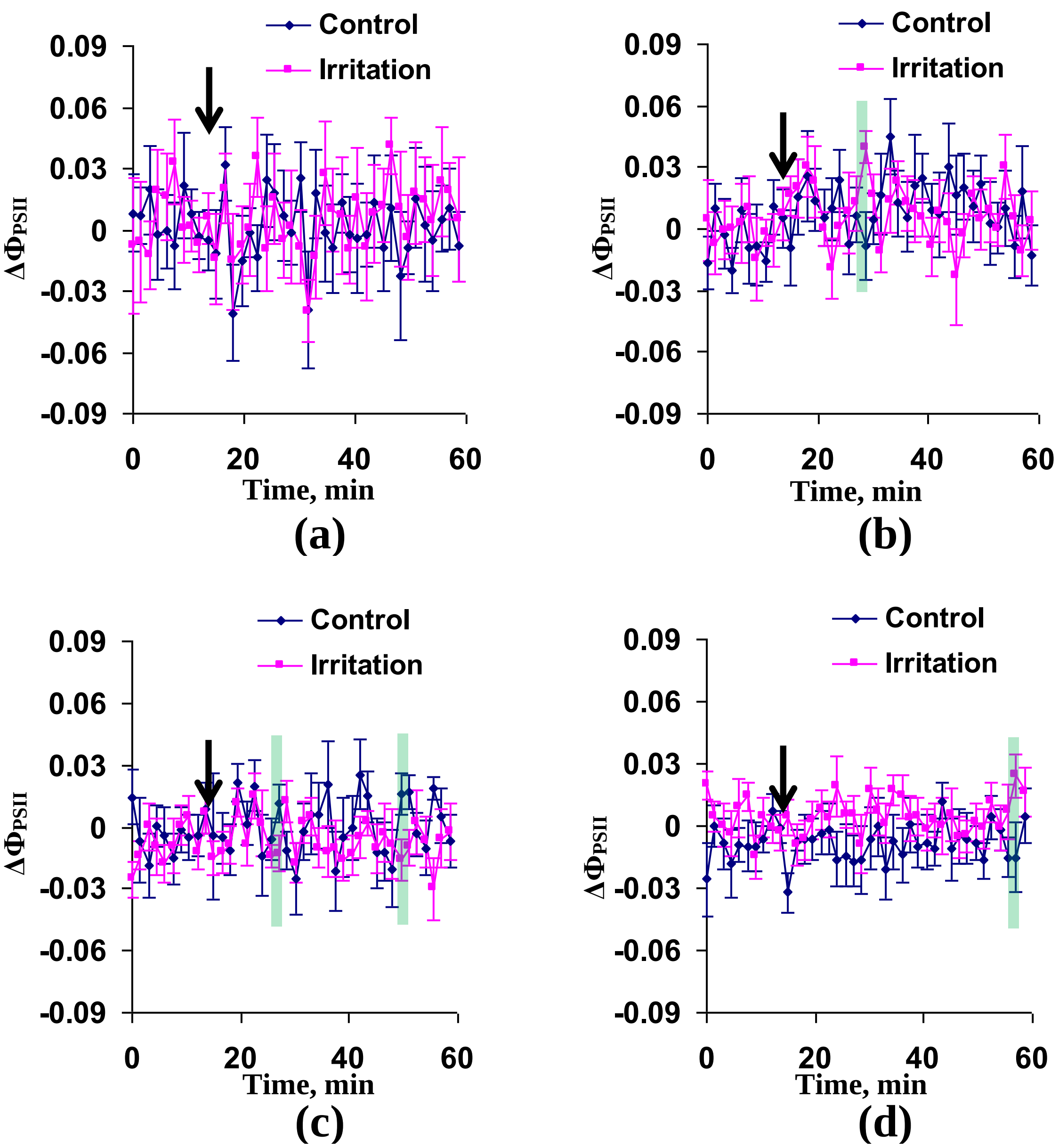

Figure S7. Dynamics of average changes in the quantum yield of photosystem II ($\Delta\Phi_{PSII}$) on 3 **(a)**, 5 **(b)**, 7 **(c)**, and 9 **(d)** cm distances from the irritated zone in wheat plants after 13-14 days of drought (n=12). Combination of local moderate heating and illumination of top of the second leaf was used for irritation (arrow). Drought was induced by termination of irrigation. Plants were cultivated for 14 days before initiation of the drought. Green shading shows significant differences between the experimental and control values (p<0.05).

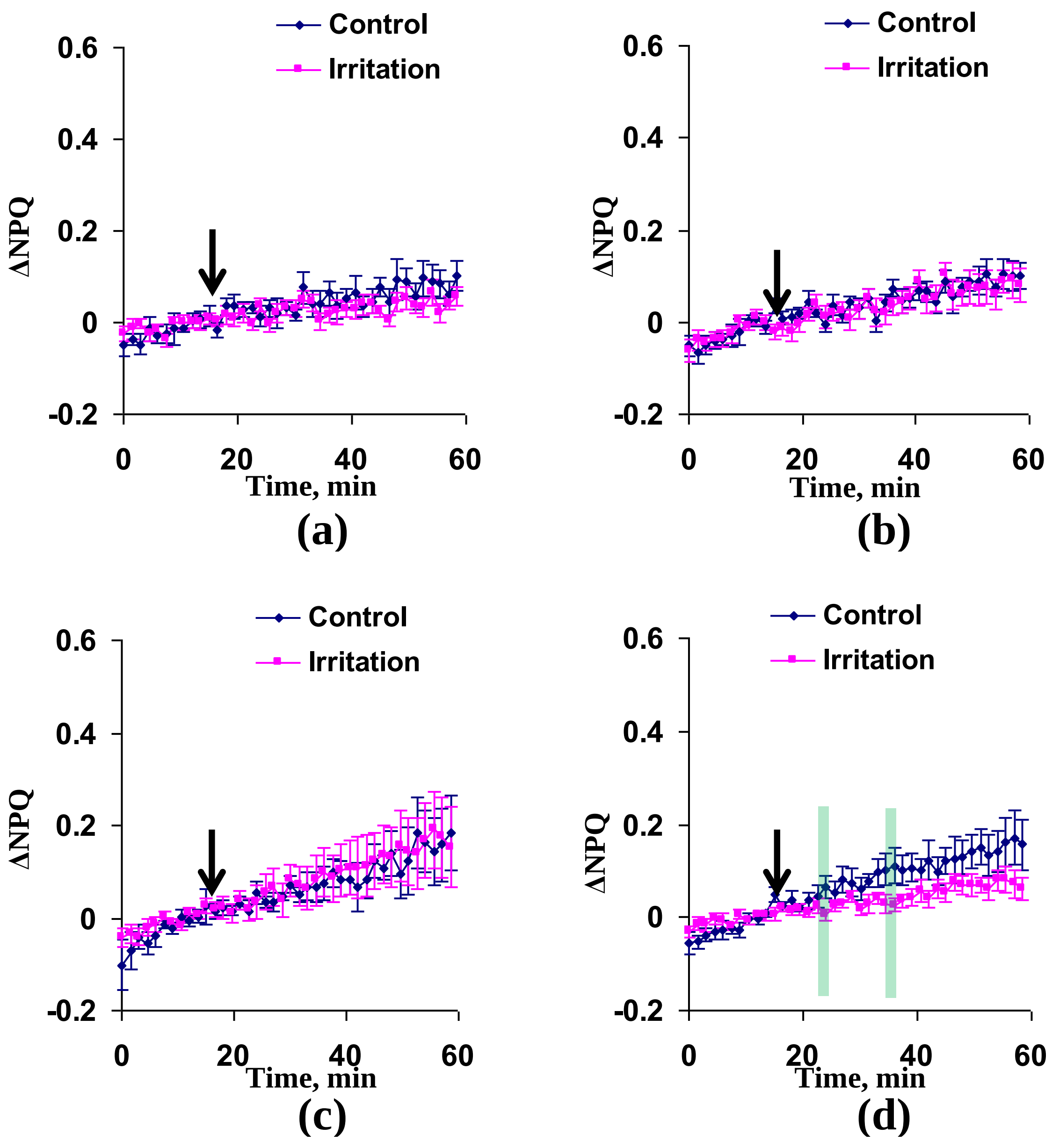

Figure S8. Dynamics of average changes in the non-photochemical quenching of chlorophyll fluorescence (ΔNPQ) on 3 **(a)**, 5 **(b)**, 7 **(c)**, and 9 **(d)** cm distances from the irritated zone in wheat plants after 13-14 days of drought (*n*=12). Combination of local moderate heating and illumination of top of the second leaf was used for irritation (arrow). Drought was induced by termination of irrigation. Plants were cultivated for 14 days before initiation of the drought. Green shading shows significant differences between the experimental and control values ($p<0.05$).
